# Supplementary material for: Addressing hurdles in cultured meat by exploring reduced myogenesis after bovine myoblast expansion
Source: Commun Biol. 2025 Nov 27;8:1851. doi: 10.1038/s42003-025-09180-8 (PMC12748566; doi:10.1038/s42003-025-09180-8)

Supplementary Tables

Supplementary Table 1. Bovine donor information.

Donor	Age category	Sex	Age
F1	Fetal	Male	4.9 gestational months
F2	Fetal	Male	6.2 gestational months
F3	Fetal	Male	7.6 gestational months
F4	Fetal	Female	7.4 gestational months
F5	Fetal	Male	5.1 gestational months
A1	Adult	Female	4 years 6.5 months
A2	Adult	Female	4 years 7 months
A3	Adult	Female	6 years 5.7 months
A4	Adult	Male	4 years 4 months
A5	Adult	Female	3 years 9 months

Supplementary Table 2. Composition of expansion media.

**PM** (Proliferation Medium)		
**Component**	**Reference**	**Final concentration**
DMEM High Glucose	Gibco, 31966-021	80%
FBS		20%
Gentamicin	Gibco, 15750037	50 µg/ml

**PM+** (Proliferation Medium +)		
**Component**	**Reference**	**Final concentration**
DMEM High Glucose	Gibco, 31966-021	80%
FBS		20%
Gentamicin	Gibco, 15750037	50 µg/ml
bFGF	Peprotech , 100-18B	5 ng/ml
p38 MAPKi	Santa Cruz, sc-204159	10 µM

**UM** (Ultroser Medium)		
**Component**	**Reference**	**Final concentration**
DMEM High Glucose	Gibco, 31966-021	89%
FBS		10%
Ultroser	Sartorius, 15950-017	1%
Gentamicin	Gibco, 15750037	50 µg/ml

**E8** (Essential 8)		
**Component**	**Reference**	**Final concentration**
Essential 8 basal medium	Gibco, A1517001	98%
Essential 8 supplement		2%

**E8 + BSA**		
**Component**	**Reference**	**Final concentration**
E8	Gibco, A1517001	99%
BSA	Sigma, A2153	1%

Supplementary Table 3. Composition of differentiation media.

**FM** (Fusion Medium)		
**Component**	**Reference**	**Final concentration**
DMEM High Glucose	Gibco, 31966-021	98%
Horse serum	Gibco, 16050130	2%
Gentamicin	Gibco, 15750037	50 µg/ml
**SkFM** (Skeletal muscle Fusion Medium)		
**Component**	**Reference**	**Final concentration**
DMEM High Glucose	Gibco, 31966-021	99.90%
BSA	Sigma, A2153	50 µg/ml
hEGF	Peprotech, AF-100-15	10 ng/ml
Insulin	BioConnect, sc-360248	10 µg/ml
Gentamicin	Gibco, 15750037	50 µg/ml

**NL15**		
**Component**	**Reference**	**Final concentration**
Neurobasal medium	Invitrogen, 21103049	50%
L15 medium	Invitrogen, 11415064	50%
hEGF	Peprotech, AF-100-15	100 ng/ml
IGF	Peprotech, 100-11	100 ng/ml

**SFDM** (Serum Free Differentiation Medium)		
**Component**	**Reference**	**Final concentration**
DMEM/F-12	Gibco, 21311-020	94.83%
hEGF	Peprotech, AF-100-15	10 ng/ml
rhAlbumin	Sigma, A9731	0.5 mg/ml
ITSE	Gibco, 51500056	2%
L-ascorbic acid 2-phosphate	Sigma, A8960	40 µM
LPA	Sigma, L7260	1 µM
MEM AA solution	Gibco, 11130036	0.5%
NaHCO3	Fluka, 88208	6.5 mM
Gentamicin	Gibco, 15750037	50 µg/ml
Soy hydrolysates	Merck, 58903C	1%

Supplementary Table 4. Overrepresentation analysis of significantly upregulated genes in high fusion index myoblasts using g:Profiler with rrvgo grouping.

rrvgo grouping	GO ID	GO term	Number of genes	q-value
**muscle structure development**	GO:0007517	muscle organ development	67	1.05E-20
	GO:0007519	skeletal muscle tissue development	38	1.21E-14
	GO:0010830	regulation of myotube differentiation	6	0.003126
	GO:0014866	skeletal myofibril assembly	2	0.016835
	GO:0014902	myotube differentiation	25	4.81E-08
	GO:0014904	myotube cell development	10	0.000156
	GO:0030239	myofibril assembly	15	2.92E-18
	GO:0030240	skeletal muscle thin filament assembly	2	0.01057
	GO:0042692	muscle cell differentiation	68	1.46E-22
	GO:0045214	sarcomere organization	10	6.25E-12
	GO:0045445	myoblast differentiation	17	0.010202
	GO:0045661	regulation of myoblast differentiation	13	0.011196
	GO:0048630	skeletal muscle tissue growth	5	0.000128
	GO:0048741	skeletal muscle fiber development	9	7.76E-05
	GO:0048742	regulation of skeletal muscle fiber development	2	0.035749
	GO:0048743	positive regulation of skeletal muscle fiber development	2	0.016835
	GO:0051146	striated muscle cell differentiation	49	1.79E-23
	GO:0051147	regulation of muscle cell differentiation	18	0.000227
	GO:0051149	positive regulation of muscle cell differentiation	12	0.008909
	GO:0051153	regulation of striated muscle cell differentiation	11	0.002533
	GO:0051155	positive regulation of striated muscle cell differentiation	10	0.001968
	GO:0055001	muscle cell development	35	9.18E-22
	GO:0055002	striated muscle cell development	15	5.42E-18
	GO:0060415	muscle tissue morphogenesis	10	0.04281
	GO:0060538	skeletal muscle organ development	38	2.89E-14
	GO:0061061	muscle structure development	116	1.58E-31
**muscle system process**	GO:0003008	system process	335	5.65E-08
	GO:0003012	muscle system process	85	9.81E-23
	GO:0003013	circulatory system process	108	3.03E-05
	GO:0003015	heart process	50	6.62E-06
	GO:0006936	muscle contraction	63	3.09E-22
	GO:0006937	regulation of muscle contraction	39	3.86E-08
	GO:0006941	striated muscle contraction	26	4.15E-17
	GO:0006942	regulation of striated muscle contraction	16	0.002533
	GO:0008015	blood circulation	101	0.000116
	GO:0008016	regulation of heart contraction	39	0.001662
	GO:0014733	regulation of skeletal muscle adaptation	4	0.00038
	GO:0014883	transition between fast and slow fiber	3	0.000128
	GO:0014888	striated muscle adaptation	11	3.5E-05
	GO:0043500	muscle adaptation	26	0.002291
	GO:0043501	skeletal muscle adaptation	7	3.19E-05
	GO:0043502	regulation of muscle adaptation	19	0.007912
	GO:0044057	regulation of system process	106	2.3E-06
	GO:0050905	neuromuscular process	24	4.18E-08
	GO:0060047	heart contraction	48	5.2E-05
	GO:0060048	cardiac muscle contraction	21	0.000107
	GO:0090257	regulation of muscle system process	53	5.46E-08
	GO:1903522	regulation of blood circulation	47	0.00977
**muscle tissue development**	GO:0009888	tissue development	350	1.87E-07
	GO:0014706	striated muscle tissue development	35	0.000141
	GO:0048641	regulation of skeletal muscle tissue development	7	4.57E-06
	GO:0048643	positive regulation of skeletal muscle tissue development	6	6.52E-05
	GO:0048738	cardiac muscle tissue development	31	6.26E-05
	GO:0055007	cardiac muscle cell differentiation	15	0.011009

	GO:0060537	muscle tissue development	75	4.37E-19
	GO:1901861	regulation of muscle tissue development	11	6.17E-06
	GO:1901863	positive regulation of muscle tissue development	7	4.57E-06
**musculoskeletal movement**	GO:0003009	skeletal muscle contraction	8	3.30266E-14
	GO:0003010	voluntary skeletal muscle contraction	2	2.76628E-05
	GO:0014721	twitch skeletal muscle contraction	2	2.76628E-05
	GO:0014724	regulation of twitch skeletal muscle contraction	2	0.001224592
	GO:0014819	regulation of skeletal muscle contraction	4	0.001274945
	GO:0050879	multicellular organismal movement	10	2.3075E-14
	GO:0050881	musculoskeletal movement	10	1.65675E-14
**cellular component assembly involved in morphogenesis**	GO:0009653	anatomical structure morphogenesis	472	6.72963E-06
	GO:0010927	cellular component assembly involved in morphogenesis	27	4.91939E-14
	GO:0030154	cell differentiation	761	1.55978E-06
	GO:0032502	developmental process	1167	4.27001E-06
	GO:0032989	cellular component morphogenesis	114	1.93597E-05
	GO:0048468	cell development	468	2.3125E-06
	GO:0048513	animal organ development	498	2.09391E-07
	GO:0048646	anatomical structure formation involved in morphogenesis	210	1.66863E-06
	GO:0048856	anatomical structure development	1046	6.56077E-07
	GO:0048869	cellular developmental process	761	1.55978E-06
**actomyosin structure organization**	GO:0007015	actin filament organization	79	0.000945795
	GO:0030029	actin filament-based process	138	2.09639E-12
	GO:0030036	actin cytoskeleton organization	125	1.25229E-09
	GO:0030048	actin filament-based movement	16	0.0017751
	GO:0030049	muscle filament sliding	2	0.0060675
	GO:0031032	actomyosin structure organization	37	3.38237E-13
	GO:0033275	actin-myosin filament sliding	2	6.42368E-05
	GO:0070252	actin-mediated cell contraction	15	0.00020277
**cytoskeleton organization**	GO:0006996	organelle organization	790	0.045890153
	GO:0007010	cytoskeleton organization	276	2.26451E-08
	GO:0030042	actin filament depolymerization	13	0.018942153
	GO:0030834	regulation of actin filament depolymerization	10	0.012499812
	GO:0030837	negative regulation of actin filament polymerization	12	0.033151428
	GO:0051694	pointed-end actin filament capping	2	0.010569948
	GO:0070925	organelle assembly	200	0.018158025
	GO:0097435	supramolecular fiber organization	153	1.83734E-07
**multicellular organismal process**	GO:0032501	multicellular organismal process	1355	2.76312E-06
**heart development**	GO:0007507	heart development	80	0.000571046
	GO:0055006	cardiac cell development	11	0.030341132
	GO:0055013	cardiac muscle cell development	11	0.025277661
**monoatomic ion transport**	GO:0006811	monoatomic ion transport	297	0.003964735
	GO:0006812	monoatomic cation transport	240	0.014327919
	GO:0010959	regulation of metal ion transport	69	0.007094796
	GO:0030001	metal ion transport	179	0.00672098
	GO:0034220	monoatomic ion transmembrane transport	231	0.01123866
	GO:0034765	regulation of monoatomic ion transmembrane transport	71	0.046695076
	GO:0043269	regulation of monoatomic ion transport	98	0.03151399
	GO:1903169	regulation of calcium ion transmembrane transport	31	0.011008531
	GO:1904062	regulation of monoatomic cation transmembrane transport	54	0.018007053

**regulation of biological quality**	GO:0042391	regulation of membrane potential	64	0.034041861
	GO:0065008	regulation of biological quality	578	0.009981988

Supplementary Table 5. Gene Set Enrichment Analysis showing significantly enriched terms (q<0.05) comparing transcriptomes of low fusion index to high fusion index myoblasts according to C5 and Hallmark databases of gene sets.

Gene set	Size	NES	q-value
**C5 database**			
GOBP: CYTOPLASMIC TRANSLATION	148	2.35861	0.001317
GOBP: INTRA GOLGI VESICLE MEDIATED TRANSPORT	30	2.25997	0.012052
GOBP: ARP2 3 COMPLEX MEDIATED ACTIN NUCLEATION	41	2.164189	0.031762
GOBP: MESENCHYMAL TO EPITHELIAL TRANSITION	15	2.12728	0.039782
GOBP: REGULATION OF HISTONE MODIFICATION	39	-1.75382	0.049276
GOBP: MITOTIC DNA INTEGRITY CHECKPOINT SIGNALING	84	-1.75452	0.049326
GOBP: POSITIVE REGULATION OF ALCOHOL BIOSYNTHETIC PROCESS	15	-1.75761	0.0481
GOBP: MITOTIC CELL CYCLE PHASE TRANSITION	410	-1.75897	0.047906
GOBP: MEIOTIC CELL CYCLE	219	-1.75992	0.047915
GOBP: NUCLEAR TRANSCRIBED MRNA CATABOLIC PROCESS	126	-1.76314	0.046823
GOBP: MUSCLE CONTRACTION	280	-1.76391	0.046854
GOBP: POSITIVE REGULATION OF CELL CYCLE	282	-1.77142	0.043746
GOBP: SKELETAL MUSCLE ORGAN DEVELOPMENT	157	-1.77181	0.044008
GOBP: WATER TRANSPORT	17	-1.772	0.04438
GOBP: CELL CELL SIGNALING INVOLVED IN CARDIAC CONDUCTION	23	-1.7743	0.043823
GOBP: HOMOLOGOUS RECOMBINATION	53	-1.77538	0.043719
GOBP: MYOTUBE DIFFERENTIATION	108	-1.78362	0.040379
GOBP: MUSCLE ORGAN DEVELOPMENT	307	-1.78959	0.038103
GOBP: POSITIVE REGULATION OF DNA METABOLIC PROCESS	279	-1.79167	0.03756
GOBP: CENTRIOLE ASSEMBLY	45	-1.79602	0.036421
GOBP: REGULATION OF MITOTIC CELL CYCLE PHASE TRANSITION	306	-1.79725	0.036262
GOBP: MEIOSIS I CELL CYCLE PROCESS	96	-1.79991	0.035589
GOBP: ORGANELLE FISSION	420	-1.80004	0.035907
GOBP: REGULATION OF ALCOHOL BIOSYNTHETIC PROCESS	36	-1.80296	0.035048
GOBP: NEUTRAL LIPID BIOSYNTHETIC PROCESS	41	-1.80296	0.035412
GOBP: REGULATION OF NUCLEAR TRANSCRIBED MRNA POLY A TAIL SHORTENING	15	-1.80528	0.034907
GOBP: POSITIVE REGULATION OF DNA REPLICATION	34	-1.80543	0.03525
GOBP: NUCLEAR TRANSCRIBED MRNA POLY A TAIL SHORTENING	34	-1.80926	0.034329
GOBP: REGULATION OF DNA TEMPLATED DNA REPLICATION INITIATION	15	-1.81246	0.033294
GOBP: REGULATION OF SKELETAL MUSCLE TISSUE DEVELOPMENT	24	-1.81298	0.033527
GOBP: REGULATION OF CELL CYCLE G2 M PHASE TRANSITION	109	-1.81317	0.033822
GOBP: RNA EXPORT FROM NUCLEUS	80	-1.81421	0.033709
GOBP: PROTEIN DNA COMPLEX ASSEMBLY	184	-1.81723	0.032852
GOBP: REGULATION OF ATTACHMENT OF SPINDLE MICROTUBULES TO KINETOCHORE	21	-1.82568	0.030235
GOBP: REGULATION OF CELL CYCLE PHASE TRANSITION	394	-1.8348	0.0275
GOBP: POSITIVE REGULATION OF SKELETAL MUSCLE TISSUE DEVELOPMENT	18	-1.83577	0.027599

GOBP: NEGATIVE REGULATION OF CELL CYCLE	351	-1.83792	0.027148
GOBP: REGULATION OF CHOLESTEROL BIOSYNTHETIC PROCESS	16	-1.84094	0.026603
GOBP: PROTEIN LOCALIZATION TO CHROMATIN	51	-1.84257	0.026421
GOBP: MATURATION OF 5 8S RRNA	36	-1.84925	0.024749
GOBP: ALCOHOL BIOSYNTHETIC PROCESS	111	-1.85314	0.023851
GOBP: DNA INTEGRITY CHECKPOINT SIGNALING	129	-1.85422	0.023899
GOBP: POSITIVE REGULATION OF CELL CYCLE PHASE TRANSITION	102	-1.86002	0.022381
GOBP: MRNA EXPORT FROM NUCLEUS	63	-1.86355	0.021796
GOBP: REGULATION OF DNA RECOMBINATION	123	-1.86806	0.020967
GOBP: NEGATIVE REGULATION OF MITOTIC CELL CYCLE	216	-1.86842	0.021197
GOBP: MRNA TRANSPORT	112	-1.86843	0.021491
GOBP: TELOMERE MAINTENANCE	149	-1.87348	0.020467
GOBP: TRIGLYCERIDE BIOSYNTHETIC PROCESS	35	-1.87492	0.020388
GOBP: NEGATIVE REGULATION OF MITOTIC CELL CYCLE PHASE TRANSITION	168	-1.88285	0.018857
GOBP: ACTIN MEDIATED CELL CONTRACTION	87	-1.8876	0.017937
GOBP: REGULATION OF DNA METABOLIC PROCESS	484	-1.89187	0.017189
GOBP: REGULATION OF MITOTIC NUCLEAR DIVISION	110	-1.89487	0.016724
GOBP: ACTIN MYOSIN FILAMENT SLIDING	15	-1.89605	0.016697
GOBP: REGULATION OF SISTER CHROMATID SEGREGATION	99	-1.90126	0.015961
GOBP: RELAXATION OF MUSCLE	28	-1.90503	0.015389
GOBP: STRIATED MUSCLE ADAPTATION	43	-1.90705	0.015217
GOBP: POSITIVE REGULATION OF CHROMOSOME ORGANIZATION	105	-1.91173	0.014655
GOBP: RNA SPLICING VIA TRANSESTERIFICATION REACTIONS	282	-1.92332	0.012911
GOBP: NEGATIVE REGULATION OF GENE EXPRESSION EPIGENETIC	102	-1.92423	0.013002
GOBP: NEGATIVE REGULATION OF DNA RECOMBINATION	44	-1.92994	0.012286
GOBP: DNA TEMPLATED DNA REPLICATION MAINTENANCE OF FIDELITY	52	-1.93046	0.012442
GOBP: MULTICELLULAR ORGANISMAL MOVEMENT	49	-1.94129	0.011239
GOBP: REGULATION OF DNA REPLICATION	112	-1.94158	0.011444
GOBP: MRNA PROCESSING	445	-1.94512	0.011081
GOBP: STEROL METABOLIC PROCESS	109	-1.95121	0.010499
GOBP: NUCLEAR CHROMOSOME SEGREGATION	282	-1.95211	0.010636
GOBP: MITOTIC DNA REPLICATION	16	-1.95349	0.010647
GOBP: MITOTIC CELL CYCLE CHECKPOINT SIGNALING	137	-1.9602	0.009837
GOBP: DNA STRAND ELONGATION	36	-1.96149	0.009919
GOBP: TELOMERE ORGANIZATION	172	-1.96431	0.009757
GOBP: NEGATIVE REGULATION OF CELL CYCLE PROCESS	277	-1.9687	0.00943
GOBP: ATTACHMENT OF SPINDLE MICROTUBULES TO KINETOCHORE	49	-1.97198	0.009177
GOBP: MISMATCH REPAIR	31	-1.97506	0.008991
GOBP: CELL CYCLE CHECKPOINT SIGNALING	183	-1.9786	0.008662
GOBP: REGULATION OF CHROMOSOME ORGANIZATION	238	-1.9843	0.008215
GOBP: PROTEIN LOCALIZATION TO CHROMOSOME CENTROMERIC REGION	36	-1.99069	0.007849
GOBP: VASCULAR TRANSPORT	70	-2.00942	0.005746

GOBP: PROTEIN LOCALIZATION TO CONDENSED CHROMOSOME	15	-2.01118	0.005847
GOBP: MATURATION OF 5 8S RRNA FROM TRICISTRONIC RRNA TRANSCRIPT SSU RRNA 5 8S RRNA LSU RRNA	25	-2.01159	0.005954
GOBP: NEGATIVE REGULATION OF CHROMOSOME ORGANIZATION	90	-2.0169	0.005693
GOBP: SKELETAL MUSCLE ADAPTATION	23	-2.01971	0.00562
GOBP: CHROMOSOME SEGREGATION	381	-2.02021	0.005776
GOBP: STRIATED MUSCLE CONTRACTION	150	-2.02845	0.005247
GOBP: REGULATION OF DNA DAMAGE CHECKPOINT	24	-2.0352	0.004735
GOBP: PROTEIN LOCALIZATION TO CHROMOSOME	109	-2.0465	0.004218
GOBP: POSITIVE REGULATION OF MUSCLE TISSUE DEVELOPMENT	22	-2.04886	0.004272
GOBP: NEGATIVE REGULATION OF CHROMOSOME SEGREGATION	46	-2.05155	0.004166
GOBP: MITOTIC SISTER CHROMATID SEPARATION	59	-2.05538	0.004026
GOBP: MITOTIC SISTER CHROMATID COHESION	31	-2.06552	0.003559
GOBP: MITOTIC NUCLEAR DIVISION	259	-2.07504	0.003238
GOBP: SKELETAL MUSCLE CONTRACTION	37	-2.09484	0.002486
GOBP: DNA RECOMBINATION	294	-2.09952	0.002323
GOBP: POSITIVE REGULATION OF CELL CYCLE PROCESS	219	-2.10935	0.002181
GOBP: NEGATIVE REGULATION OF NUCLEAR DIVISION	58	-2.11133	0.002167
GOBP: SISTER CHROMATID SEGREGATION	217	-2.11982	0.001969
GOBP: DNA UNWINDING INVOLVED IN DNA REPLICATION	21	-2.12065	0.002021
GOBP: REGULATION OF CELL CYCLE CHECKPOINT	43	-2.12264	0.002036
GOBP: POSITIVE REGULATION OF CHROMOSOME SEPARATION	27	-2.1278	0.001804
GOBP: RECOMBINATIONAL REPAIR	169	-2.13316	0.001767
GOBP: DOUBLE STRAND BREAK REPAIR	288	-2.13352	0.001865
GOBP: CHROMOSOME CONDENSATION	36	-2.14224	0.001777
GOBP: REGULATION OF CHROMOSOME SEGREGATION	120	-2.14692	0.001676
GOBP: NUCLEOSOME ORGANIZATION	99	-2.15179	0.001622
GOBP: REGULATION OF MITOTIC SISTER CHROMATID SEGREGATION	51	-2.17274	0.001137
GOBP: CELL CYCLE DNA REPLICATION	42	-2.2012	7.09E-04
GOBP: DNA REPLICATION INITIATION	36	-2.21212	6.98E-04
GOBP: MITOTIC CHROMOSOME CONDENSATION	18	-2.21711	6.85E-04
GOBP: SISTER CHROMATID COHESION	54	-2.22869	7.53E-04
GOBP: REGULATION OF DNA TEMPLATED DNA REPLICATION	43	-2.25159	3.71E-04
GOBP: CHROMOSOME SEPARATION	76	-2.25722	3.14E-04
GOBP: DNA CONFORMATION CHANGE	89	-2.25796	3.59E-04
GOBP: MITOTIC SISTER CHROMATID SEGREGATION	178	-2.26054	2.79E-04
GOBP: DNA STRAND ELONGATION INVOLVED IN DNA REPLICATION	15	-2.28261	3.34E-04
GOBP: DNA REPLICATION	263	-2.29876	2.05E-04
GOBP: DNA TEMPLATED DNA REPLICATION	155	-2.38237	0
GOBP: POSITIVE REGULATION OF CHROMOSOME SEGREGATION	26	-2.39794	0
GOBP: STEROL BIOSYNTHETIC PROCESS	51	-2.49033	0
**Hallmark database**			
HM: PROTEIN SECRETION	91	2.0038	0

HM: EPITHELIAL MESENCHYMAL TRANSITION	189	1.7453	0.013785676
HM: INTERFERON ALPHA RESPONSE	86	-1.4537	0.04800727
HM: KRAS SIGNALING DN	130	-1.4796	0.043305725
HM: CHOLESTEROL HOMEOSTASIS	68	-1.5042	0.03898992
HM: MITOTIC SPINDLE	196	-1.6256	0.013980814
HM: MYC TARGETS V2	56	-1.7764	0.003479767
HM: MYOGENESIS	193	-2.2754	0
HM: G2M CHECKPOINT	195	-2.7112	0
HM: E2F TARGETS	197	-2.7512	0

GOPB: Gene Ontology Biological Process
HM: Hallmark
NES: Normalized Enrichment Score

Supplementary Table 6. Ingenuity Pathway Analysis showing significantly affected pathways (q<0.05, |z-score|>0) comparing transcriptomes of low fusion index to high fusion index myoblasts.

Ingenuity Canonical Pathways	-log(p-value)	Ratio	Z-score	Molecules
RHOGDI Signaling	2.45E00	5.48E-02	1.890	*ACTA1,ACTC1,CDH17,ITGB6,LIMK2,MYH2,MYH3,MYH7,MYL1,MYL11,MYL4,MYO18B*
HEY1 Signaling Pathway	1.89E00	5.73E-02	1.414	*DLL1,ERBB3,GATA2,JAG1,MEF2C,MFAP5,MMP28,MYOG,PRKAG2*
CLEAR Signaling Pathway	2.09E-01	1.79E-02	1.342	*CTSD,FGFR4,PRKAG2,PRKCB,TLR2*
Cachexia Signaling Pathway	7.53E-01	2.87E-02	1.265	*CAPN6,GUCY1A1,IL17B,IL1RN,MEF2C,MYOG,PRKAG2,PRKCB,RYR1,SLC2A4*
Role of Chondrocytes in Rheumatoid Arthritis Signaling Pathway	3.76E-01	2.9E-02	1.000	*IL1RN,MMP28,PRKAG2,TLR2*
DHCR24 Signaling Pathway	3.8E-01	2.92E-02	1.000	*PRKCB,SREBF1,TM7SF2,VCAM1*
Sertoli Cell-Germ Cell Junction Signaling Pathway (Enhanced)	1.87E-01	1.75E-02	1.000	*ACTA1,ACTC1,GUCY1A1,PRKAG2*
TR/RXR Activation	4.01E-01	3.23E-02	1.000	*ATP2A1,MYH7,RAB3B,SREBF1*
IL-8 Signaling	2.16E-01	1.91E-02	1.000	*ARAF,LIMK2,PRKCB,VCAM1*
Protein Kinase A Signaling	1.24E00	3.3E-02	1.000	*DUSP1,GUCY1A1,GYS1,MYH2,MYL1,MYL11,MYL4,PDE2A,PDE3A,PRKAG2,PRKCB,PTPRS,RYR1*
Sirtuin Signaling Pathway	1.22E-01	1.4E-02	1.000	*ACLY,EPAS1,SREBF1,TUBA4A*
RAR Activation	1.41E-01	1.47E-02	0.816	*DUSP1,GUCY1A1,IL17B,PDE2A,PDE3A,PRKAG2*
Corticotropin Releasing Hormone Signaling	8.15E-01	4.05E-02	0.816	*CACNG1,CACNG4,GUCY1A1,MEF2C,PRKAG2,PRKCB*
Adrenomedullin signaling pathway	5.33E-01	3.12E-02	0.816	*ARAF,GUCY1A1,IL1RN,PRKAG2,RAMP3,TFAP2A*
Neurovascular Coupling Signaling Pathway	3.18E-01	2.29E-02	0.447	*CACNG1,CACNG4,GUCY1A1,PRKAG2,RYR1*
Wound Healing Signaling Pathway	2.8E-01	2.13E-02	0.447	*COL17A1,COL18A1,IL17B,IL1RN,KRT6A*
Macrophage Alternative Activation Signaling Pathway	4.07E-01	2.92E-02	0.447	*ACLY,DUSP1,EPAS1,IL1RN,SREBF1*
Activin Inhibin Signaling Pathway	3.69E-01	2.58E-02	0.447	*GATA2,IL1RN,LIMK2,PAX2,TLR2*
GABAergic Receptor Signaling Pathway (Enhanced)	5.98E-01	3.68E-02	0.447	*CACNG1,CACNG4,GUCY1A1,PRKAG2,SLC6A13*
FXR/RXR Activation	4.01E-01	2.86E-02	0.447	*FGFR4,IL17B,IL1RN,PRKAG2,SREBF1*
HIF1α Signaling	3.47E-01	2.45E-02	0.447	*ARAF,IGF2,MMP28,PRKCB,SLC2A4*
Human Embryonic Stem Cell Pluripotency	3.55E-01	2.53E-02	0.447	*ARAF,FGFR4,FZD2,PRKCB,SPHK1*
NF-κB Signaling	3.23E-01	2.31E-02	0.447	*ARAF,FGFR4,IL1RN,PRKCB,TLR2*
GPCR-Mediated Nutrient Sensing in Enteroendocrine Cells	1.09E00	5.17E-02	0.447	*CACNG1,CACNG4,GPBAR1,GUCY1A1,PRKAG2,PRKCB*
Senescence Pathway	5.56E-01	2.78E-02	0.447	*ARAF,CACNG1,CACNG4,CAPN6,CAT,CCNE2,RASSF5,TLR2*
Xenobiotic Metabolism PXR Signaling Pathway	4.01E-01	2.86E-02	0.447	*CAT,PRKAG2,PRKCB,SMOX,SULT4A1*
Insulin Secretion Signaling Pathway	6.43E-01	0.03	0.447	*ABCC9,CACNG1,CACNG4,GUCY1A1,PRKAG2,PRKCB,RYR1,SLC2A4*

Pulmonary Healing Signaling Pathway	8.03E-01	3.55E-02	0.378	*ARAF,FZD2,JAG1,MMP28,PRKAG2,PRKCB,TLR2*
Adrenergic Receptor Signaling Pathway (Enhanced)	8.24E-01	3.7E-02	0.378	*CACNG1,CACNG4,GUCY1A1,IL17B,PRKAG2,PRKCB,SMOX*
Opioid Signaling Pathway	8.6E-01	3.31E-02	0.378	*CACNG1,CACNG4,GUCY1A1,PRKAG2,PRKCB,RGS14,RGS16,RYR1,SCN7A*
BBSome Signaling Pathway	1.59E-01	1.52E-02	-0.378	*FZD2,GPR65,GPRC5C,LPAR3,MYL11,NPY1R,PRKAG2*
Gustation Pathway	1.09E00	4.15E-02	-0.378	*ABCC9,CACNG1,CACNG4,GUCY1A1,PDE3A,PRKAG2,SCN7A,SLC2A4*
RAF/MAP kinase cascade	2.43E-01	1.92E-02	-0.447	*ACTN2,ARAF,DUSP1,ERBB3,FGFR4*
Pancreatic Secretion Signaling Pathway	2.78E-01	2.07E-02	-0.447	*ATP2A1,GUCY1A1,PRKAG2,PRKCB,RYR1*
WNT/SHH Axonal Guidance Signaling Pathway	5.23E-01	3.42E-02	-0.447	*FZD2,GUCY1A1,PLXNA1,PRKAG2,SHISA2*
HER-2 Signaling in Breast Cancer	2.92E-01	2.23E-02	-0.447	*ARAF,CCNE2,ERBB3,ITGB6,PRKCB*
Role of Macrophages, Fibroblasts and Endothelial Cells in Rheumatoid Arthritis	1.65E-01	1.6E-02	-0.447	*FZD2,IL1RN,PRKCB,TLR2,VCAM1*
Netrin Signaling	4.15E-01	2.96E-02	-0.447	*CACNG1,CACNG4,PRKAG2,PRKCB,RYR1*
D-myo-inositol-5-phosphate Metabolism	3.69E-01	2.58E-02	-0.447	*DUSP1,DUSP13B,NUDT4,PPP1R1A,STYXL2*
D-myo-inositol (1,4,5,6)-Tetrakisphosphate Biosynthesis	3.94E-01	2.81E-02	-0.447	*DUSP1,DUSP13B,NUDT4,PPP1R1A,STYXL2*
Superpathway of Inositol Phosphate Compounds	2.85E-01	2.16E-02	-0.447	*DUSP1,DUSP13B,NUDT4,PPP1R1A,STYXL2*
D-myo-inositol (3,4,5,6)-tetrakisphosphate Biosynthesis	3.94E-01	2.81E-02	-0.447	*DUSP1,DUSP13B,NUDT4,PPP1R1A,STYXL2*
3-phosphoinositide Degradation	3.8E-01	2.65E-02	-0.447	*DUSP1,DUSP13B,NUDT4,PPP1R1A,STYXL2*
3-phosphoinositide Biosynthesis	3.47E-01	2.45E-02	-0.447	*DUSP1,DUSP13B,NUDT4,PPP1R1A,STYXL2*
S100 Family Signaling Pathway	3.8E-01	1.94E-02	-0.535	*ARAF,CACNG1,CACNG4,CTSD,FGFR4,FZD2,GPR65,GPRC5C,LPAR3,MMP28,NPY1R,PRKAG2,PRKCB,VCAM1*
CREB Signaling in Neurons	3.32E-01	1.91E-02	-0.632	*CACNG1,CACNG4,FGFR4,FZD2,GPR65,GPRC5C,GUCY1A1,LPAR3,NPY1R,PRKAG2,PRKCB*
Leukocyte Extravasation Signaling	1.13E00	4.23E-02	-0.707	*ACTA1,ACTC1,ACTN2,ACTN3,MMP28,PRKCB,RASSF5,VCAM1*
G-Protein Coupled Receptor Signaling	8.91E-01	2.53E-02	-0.728	*DUSP1,FZD2,GPR65,GPRC5C,GUCY1A1,LPAR3,MEF2C,MYL1,MYL11,MYL4,NPY1R,PDE2A,PDE3A,PRKAG2,PRKCB,RGS14,RGS16*
Glutaminergic Receptor Signaling Pathway (Enhanced)	2.55E-01	1.89E-02	-0.816	*CACNG1,CACNG4,GUCY1A1,PRKAG2,PRKCB,SCN7A*
Orexin Signaling Pathway	3.94E-01	2.61E-02	-0.816	*CACNG1,CACNG4,GUCY1A1,PRKAG2,PRKCB,SLC2A4*
White Adipose Tissue Browning Pathway	8.91E-01	4.44E-02	-0.816	*CACNG1,CACNG4,FGFR4,GPBAR1,GUCY1A1,PRKAG2*
ABRA Signaling Pathway	3.35E00	0.1	-1.000	*ACTA1,ACTC1,CKM,CKMT2,LIMK2,MEF2C,MYL1,MYOG,SVIL*
Extracellular matrix organization	4.94E-01	3.77E-02	-1.000	*COL18A1,ITGB6,MUSK,PTPRS*
Signaling by NOTCH1	7.56E-01	5.19E-02	-1.000	*DLL1,JAG1,MAMLD1,NEURL1*
Signaling by NOTCH2	1.59E00	1.21E-01	-1.000	*DLL1,JAG1,MAMLD1,NEURL1*

G alpha (q) signalling events	2.85E-01	2.37E-02	-1.000	*GPR65,LPAR3,OXT,RGS16*
Response to elevated platelet cytosolic Ca2+	3.8E-01	3.03E-02	-1.000	*ACTN2,IGF2,PRKCB,TUBA4A*
Signaling by NOTCH3	1.13E00	8.16E-02	-1.000	*DLL1,JAG1,MAMLD1,NEURL1*
CXCR4 Signaling	4.17E-01	2.99E-02	-1.000	*GUCY1A1,MYL1,MYL11,MYL4,PRKCB*
Virus Entry via Endocytic Pathways	4.24E-01	3.42E-02	-1.000	*ACTA1,ACTC1,ITGB6,PRKCB*
CDC42 Signaling	3.8E-01	2.66E-02	-1.000	*ITGB6,LIMK2,MYL1,MYL11,MYL4*
PAK Signaling	7.53E-01	4.27E-02	-1.000	*ITGB6,LIMK2,MYL1,MYL11,MYL4*
Oxytocin in Brain Signaling Pathway	3.67E-01	2.56E-02	-1.342	*CACNG1,CACNG4,MEF2C,OXT,PRKCB*
Synaptic Long Term Depression	3.75E-01	2.6E-02	-1.342	*CACNG1,CACNG4,GUCY1A1,PRKCB,RYR1*
Phospholipase C Signaling	2.78E-01	1.93E-02	-1.342	*GUCY1A1,ITGB6,MEF2C,MYL1,MYL11,MYL4,PRKCB*
Adipogenesis pathway	5.98E-01	3.7E-02	-1.342	*AGPAT2,FGFR4,FZD2,SLC2A4,SREBF1*
Phagosome Formation	8.07E-01	2.47E-02	-1.500	*FZD2,GPR65,GPRC5C,ITGB6,LIMK2,LPAR3,MYH2,MYH3,MYH7,MYL1,MYL4,MYO18B,NPY1R,PRKCB,SPHK1,TLR2*
NAD Signaling Pathway	8.39E-01	4.17E-02	-.,633	*NMRK2,OXT,PRKAG2,RYR1,SLC29A1,SREBF1*
Class A/1 (Rhodopsin-like receptors)	2.8E-01	2.02E-02	-1.633	*CXCL10,CXCL9,GPR65,LPAR3,NPY1R,OXT*
NAFLD Signaling Pathway	4.37E-01	2.8E-02	-1.633	*GYS1,IL17B,PRKAG2,SCD,SREBF1,TLR2*
Gα12/13 Signaling	8.94E-01	4.51E-02	-1.633	*CDH17,LPAR3,MEF2C,MYL1,MYL11,MYL4*
Ephrin A Signaling	8.91E-01	4.38E-02	-1.633	*ARAF,EPAS1,MYL1,MYL11,MYL4,PRKAG2*
Pathogen Induced Cytokine Storm Signaling Pathway	5.98E-01	2.74E-02	-1.667	*COL17A1,COL18A1,CXCL10,CXCL9,IL17B,IL1RN,RYR1,SLC2A4,TLR2*
Myelination Signaling Pathway	6.36E-01	2.82E-02	-1.667	*ARAF,ERBB3,FGFR4,FZD2,ITGB6,PRKAG2,SCD,SOX8,SREBF1*
Acetylcholine Receptor Signaling Pathway	1.56E00	4.79E-02	-1.667	*CACNG1,CACNG4,CHRNA1,CHRNB1,CHRND,CHRNG,GUCY1A1,PRKAG2,PRKCB*
Autism Signaling Pathway	3.8E-01	2.36E-02	-1.890	*CACNG1,CACNG4,FZD2,IGF2,IL17B,IL1RN,PRKAG2*
RHOA Signaling	1.92E00	6.56E-02	-1.890	*ACTA1,ACTC1,LIMK2,LPAR3,MYL1,MYL11,MYL4,PLXNA1*
Gap Junction Signaling	3.54E-01	2.24E-02	-1.890	*ACTA1,ACTC1,GUCY1A1,OXT,PRKAG2,PRKCB,TUBA4A*
ID1 Signaling Pathway	1.81E00	5.05E-02	-1.897	*ARAF,CDKN1C,CHRNA1,CHRNB1,CHRND,CHRNG,FGFR4,IGF2,SEMA4C,TFAP2A*
Multiple Sclerosis Signaling Pathway	2.26E-01	1.95E-02	-2.000	*CAPN6,DUSP1,IL17B,TLR2*
Cell junction organization	5.98E-01	4.4E-02	-2.000	*CDH17,COL17A1,SDK1,SDK2*
Agrin Interactions at Neuromuscular Junction	1.3E00	7.25E-02	-2.000	*ACTA1,ACTC1,CHRNA1,ERBB3,MUSK*
Paxillin Signaling	8.18E-01	4.67E-02	-2.000	*ACTA1,ACTC1,ACTN2,ACTN3,ITGB6*
nNOS Signaling in Skeletal Muscle Cells	1.16E00	8.51E-02	-2.000	*CACNG1,CACNG4,CHRNA1,RYR1*
SNARE Signaling Pathway	1.76E00	6.02E-02	-2.121	*GUCY1A1,MYH2,MYH3,MYH7,MYL1,MYL4,MYO18B,PRKAG2*
Signaling by Rho Family GTPases	8.91E-01	3.38E-02	-2.121	*ACTA1,ACTC1,CDH17,DES,ITGB6,LIMK2,MYL1,MYL11,MYL4*
Regulation of Actin-based Motility by Rho	1.13E00	5.36E-02	-2.236	*ACTA1,ACTC1,ITGB6,MYL1,MYL11,MYL4*
VEGF Signaling	8.91E-01	5.05E-02	-2.236	*ACTA1,ACTC1,ACTN2,ACTN3,PRKCB*

G alpha (i) signalling events	3.8E-01	2.46E-02	-2.449	*CXCL10,CXCL9,LPAR3,NPY1R,RGS14,RGS16*
Nuclear Cytoskeleton Signaling Pathway	4.22E-01	2.76E-02	-2.449	*ACTA1,ACTC1,CDH17,DES,ITGB6,TUBA4A*
Sertoli Cell-Sertoli Cell Junction Signaling	3.8E-01	2.5E-02	-2.449	*ACTA1,ACTC1,ACTN2,ACTN3,CDH17,TUBA4A*
Integrin Signaling	9.73E-01	3.85E-02	-2.449	*ACTA1,ACTC1,ACTN2,ACTN3,CAPN6,ITGB6,NEDD9,TSPAN7*
Calcium Signaling	1.23E01	1.11E-01	-2.496	*ACTA1,ACTC1,ATP2A1,CACNG1,CACNG4,CASQ2,CHRNA1,CHRNB1,CHRND,CHRNG,MEF2C,MYH2,MYH3,MYH7,MYL1,MYL4,MYO18B,PRKAG2,RYR1,TNNC1,TNNC2,TNNT1,TNNT2,TNNT3*
Oxytocin Signaling Pathway	2.63E00	5.11E-02	-2.673	*ABCC9,CACNG1,CACNG4,GUCY1A1,MEF2C,MYH2,MYH3,MYH7,MYL1,MYL4,MYO18B,OXT,PRKAG2,PRKCB*
Actin Cytoskeleton Signaling	2.63E00	5.39E-02	-2.714	*ACTA1,ACTC1,ACTN2,ACTN3,ITGB6,LIMK2,MYH2,MYH3,MYH7,MYL1,MYL11,MYL4,MYO18B*
ILK Signaling	2.35E00	5.56E-02	-3.162	*ACTA1,ACTC1,ACTN2,ACTN3,ITGB6,MYH2,MYH3,MYH7,MYL1,MYL4,MYO18B*
Striated Muscle Contraction	1.58E01	4.17E-01	-3.873	*ACTA1,ACTC1,ACTN2,ACTN3,DES,MYH3,MYL1,MYL4,NEB,TMOD1,TNNC1,TNNC2,TNNT1,TNNT2,TNNT3*

Supplementary Table 7. Cluster of differentially expressed peptides between low fusion index and high fusion index myoblasts used for subsequent analyses.

Peptide	log2FC	q-value	Protein	Gene
QLEEKESTVSQLSR	-2.83	0.026	A6QPA6	*MYH3*
VSDLTQAANKNNDALR	-2.58	0.049	DESM	*DES*
ETSPEQR	-2.57	0.026	DESM	*DES*
NISEAEEWYK	-2.56	0.026	DESM	*DES*
AGFGTKGSSSSVTSR	-2.47	0.026	DESM	*DES*
VTVETEDNR	-2.46	0.418	A6QPA6	*MYH3*
IKSTQDGKVTVETEDNR	-2.41	0.026	A6QPA6	*MYH3*
AQYETIAAK	-2.41	0.026	DESM	*DES*
VKVGNEYVTK	-2.39	0.050	MYH2	*MYH2*
GSEVHTKK	-2.38	0.026	DESM	*DES*
TYCFVVDSKEEYAK	-2.25	0.086	A6QPA6	*MYH3*
VHEEEIRELQAQLQEQQVQVEMDMSKPDLTAALR	-2.21	0.050	DESM	*DES*
VIQYFATIAATGDLAK	-2.19	0.109	A6QPA6	*MYH3*
SAETEKEMATMKEEFQK	-2.19	0.109	MYH2	*MYH2*
ALQEAHQQTLDDLQAEEDKVNSLSK	-2.13	0.146	A6QPA6	*MYH3*
RIESLNEEIAFLK	-2.11	0.086	DESM	*DES*
QAAETVR	-2.11	0.164	A6QPA6	*MYH3*
IKEVTERAEDEEEMNAELTAK	-2.09	0.168	A6QPA6	*MYH3*
YASGASCLATR	-2.08	0.107	A0AAF6DLZ6	*KLHL41*
SKQAFTQQIEELKR	-2.03	0.237	A6QPA6	*MYH3*
LQTEAGEYSR	-2.02	0.140	F1N775	*MYH8*
QVEVLTNQR	-1.99	0.261	DESM	*DES*
LQTEAGELSR	-1.99	0.264	A6QPA6	*MYH3*
TLEDQLSEAR	-1.94	0.347	A6QPA6	*MYH3*
NISEAEEWYKSK	-1.90	0.375	DESM	*DES*
VKNLSEVFDCIR	-1.90	0.241	A0AAF6DLZ6	*KLHL41*
NFDKVLAEWK	-1.89	0.375	A6QPA6	*MYH3*
GGPASQPR	-1.89	0.418	A0AAA9RRF7	*DDX31*
RIESLNEEIAFLKK	-1.89	0.375	DESM	*DES*
AGLLGTLEEMRDDRLAK	-1.89	0.375	A6QPA6	*MYH3*
NLTEELAGLDETIAK	-1.88	0.378	A6QPA6	*MYH3*
VDVERDNLLDDLQR	-1.88	0.261	DESM	*DES*
ALSKANSEVAQWR	-1.86	0.273	A6QPA6	*MYH3*
ALGQNPTNAEVLR	-1.82	0.495	A0AAA9SGS4	*MYL4*
TKYETDAIQR	-1.80	0.511	MYH2	*MYH2*
LQEEIQLKEEAENNLAAFR	-1.79	0.375	DESM	*DES*
KLEGDLK	-1.79	0.375	MYH2	*MYH2*
AKFQLEAK	-1.78	0.567	A6QPA6	*MYH3*
SKVSDLTQAANKNNDALR	-1.78	0.567	DESM	*DES*
LQAEVEDLMVDVDRANSLAAALDKK	-1.76	0.409	A6QPA6	*MYH3*
QAKQEMMEYR	-1.72	0.470	DESM	*DES*
QREEQAEPDGTEVADKTAYLMGLNSSDLLK	-1.69	0.532	A6QPA6	*MYH3*

LLASIDIDHTQYK	-1.65	0.645	MYH2	*MYH2*
EKSEFKLELDDLGSNVESVSK	-1.64	0.677	A6QPA6	*MYH3*
TSGGAGGLGALR	-1.64	0.677	DESM	*DES*
QLQLGER	-1.62	0.721	E1BF59	*PLEC*
HQIQSYTCEIDALKGTNDSLMR	-1.60	0.799	DESM	*DES*
VAEIYEEELR	-1.60	0.806	DESM	*DES*
ILYGDFKQR	-1.57	0.949	A6QPA6	*MYH3*
GTLEDQIISANPLLEAFGNAK	-1.56	0.967	A6QPA6	*MYH3*
LAVPIILR	1.69	0.949	FETA	*HSP90AB1*
GYKHTLNQIDSVKVWPR	1.71	0.511	FETUA	*AHSG*
LIGQIVSSITASLR	1.93	0.221	TBA1D	*TUBA1D*

Supplementary Table 8. Enrichment analyses comparing proteomes of low fusion index to high fusion index myoblasts using different tools.

Category	Term ID	Term description	Observed gene count	Background gene count	q-value
GO Process	GO:0006936	Muscle contraction	4	149	0.0094
GO Process	GO:0006941	Striated muscle contraction	3	75	0.0303
GO Function	GO:0003779	Actin binding	5	391	0.0031
GO Function	GO:0008092	Cytoskeletal protein binding	6	877	0.0032
GO Function	GO:0051015	Actin filament binding	4	205	0.0033
GO Function	GO:0005200	Structural constituent of cytoskeleton	3	59	0.0034
GO Function	GO:0003774	Cytoskeletal motor activity	3	108	0.0159
GO Component	GO:0030016	Myofibril	7	170	1.13E-09
GO Component	GO:0099512	Supramolecular fiber	8	799	2.89E-07
GO Component	GO:0030017	Sarcomere	5	147	2.22E-06
GO Component	GO:0016459	Myosin complex	4	54	3.61E-06
GO Component	GO:0032982	Myosin filament	3	20	3.63E-05
GO Component	GO:0016460	Myosin II complex	3	24	5.33E-05
GO Component	GO:0005856	Cytoskeleton	8	1991	0.00011
GO Component	GO:0043232	Intracellular non-membrane-bounded organelle	9	4267	0.0025
GO Component	GO:0031672	A band	2	23	0.0085
GO Component	GO:0005737	Cytoplasm	11	10284	0.0291
STRING clusters	CL:22366	Mixed, incl. Myofibril, and Smooth Muscle Contraction	4	121	0.0016
STRING clusters	CL:22376	Striated muscle contraction, and Thick filament	3	30	0.0016
STRING clusters	CL:22381	Troponin complex, and DNA repair protein XRCC4-like, C-terminal	2	12	0.0091
Reactome	BTA-390522	Striated Muscle Contraction	4	27	1.75E-06
WikiPathways	WP969	Striated muscle contraction	4	34	6.08E-07
TISSUES	BTO:0001651	Longissimus thoracis	3	27	0.00084
TISSUES	BTO:0000887	Muscle	4	421	0.0184
COMPARTMENTS	GOCC:0030016	Myofibril	6	133	5.09E-08
COMPARTMENTS	GOCC:0030017	Sarcomere	4	106	7.45E-05
COMPARTMENTS	GOCC:0016460	Myosin II complex	2	13	0.0066
COMPARTMENTS	GOCC:0043232	Intracellular non-membrane-bounded organelle	7	2813	0.0289
UniProt Keywords	KW-0009	Actin-binding	4	150	0.00038
UniProt Keywords	KW-0518	Myosin	3	43	0.00038
UniProt Keywords	KW-0514	Muscle protein	2	25	0.01
UniProt Keywords	KW-0547	Nucleotide-binding	5	1246	0.021
UniProt Keywords	KW-0488	Methylation	3	310	0.0367
Pfam	PF01576	Myosin tail	3	18	0.00015
Pfam	PF02736	Myosin N-terminal SH3-like domain	3	14	0.00015
InterPro	IPR014751	DNA repair protein XRCC4-like, C-terminal	3	11	0.00025
InterPro	IPR008989	Myosin S1 fragment, N-terminal	3	15	0.00028

InterPro	IPR002928	Myosin tail	3	18	0.00031
InterPro	IPR004009	Myosin, N-terminal, SH3-like	3	18	0.00031
InterPro	IPR001609	Myosin head, motor domain	3	36	0.0013
InterPro	IPR000048	IQ motif, EF-hand binding site	3	81	0.0108
InterPro	IPR036961	Kinesin motor domain superfamily	3	81	0.0108
SMART	SM00242	Myosin. Large ATPases.	3	35	0.00064
SMART	SM00015	Short calmodulin-binding motif containing conserved Ile and Gln residues.	3	83	0.0038

Supplementary Table 9. Expression changes of the DEG, which are positively correlated with FI in the whole dataset (Spearman's R > 0.6, n=10), comparing P1 to P3 for the A1 sample. The A1 sample is of particular interest, because the FI was exceptionally preserved in A1 up to P6. Not all DEG that are positively correlated with FI in the whole dataset are preserved in the A1 sample during expansion. Preserved is defined here as fold change ≥ 0.8 from P1 to P3. Some of these DEG even display a decreased expression, defined here as fold change < 0.8 from P1 to P3.

Gene	Full name	Counts in P1	Counts in P3	Spearman's correlation of FI to counts in the whole dataset	q-value of Spearman's correlation of FI to counts
**Genes with preserved or increased expression in A1 sample from P1 to P3**					
PRG4	Proteoglycan 4	36	62	0.611	0.091
PSTPIP1	Proline-serine-threonine phosphatase-interacting protein 1	11	14	0.768	0.029
VCAM1	Vascular cell adhesion protein 1	8	38	0.950	0.002
RAMP3	Receptor activity modifying protein 3	92	275	0.743	0.036
KCNAB1	Voltage gated potassium channel subunit beta-1	15	43	0.912	0.005
KCNE4	Potassium voltage-gated channel subfamily E member 4	241	193	0.872	0.013
SCG2	Secretogranin II (chromogranin C)	54	77	0.756	0.031
OXT	Oxytocin	191	144	0.866	0.013
TLR2	Toll-like receptor 2	115	112	0.640	0.074
RGS16	Regulator of G-protein signaling 16	427	555	0.909	0.005
SFMBT2	Scm Like With Four Mbt Domains 2	71	61	0.671	0.061
NPY1R	Neuropeptide Y receptor Y1	35	30	0.963	0.001
CDH17	Cadherin-17	22	24	0.848	0.019
**Genes with decreased expression in A1 sample from P1 to P3**					
RASL10 A	Ras-like protein family member 10A	18	10	0.939	0.003
CBLN4	Cerebellin 4 precursor	778	109	0.848	0.019
NNAT	Neuronatin	1370	444	0.841	0.019
MAP4	Microtubule associated protein 4	1647	1238	0.835	0.020
NEB	Nebulin	2356	422	0.835	0.020
SDK2	Protein sidekick-2	13	2	0.833	0.020
VWA5A	Von Willebrand factor A domain containing 5A	609	225	0.829	0.021
CXCL10	C-X-C motif chemokine ligand 10	18	2	0.826	0.021
SLC2A4	Glucose transporter type 4, aka solute carrier family 2, facilitated glucose transporter member 4	64	24	0.823	0.022
RYR1	Ryanodine receptor 1	11	1	0.821	0.022
MYMK	Myomaker	21	10	0.811	0.024
MYPN	Myopalladin	27	14	0.807	0.025
BCAM	Basal cell adhesion molecule	444	228	0.805	0.025
LPAR3	Lysophosphatidic acid receptor 3	18	6	0.696	0.054

Supplementary Figures

Supplementary Figure 1. Flow cytometric gating strategies for CD56 marker analysis (**A**) and ß-galactosidase activity (**B**). A minimum of 10,000 events were measured in the "Debri- & aggregate-free cells" population. Percentages mentioned in each box represent a percentage of the respective parent population.

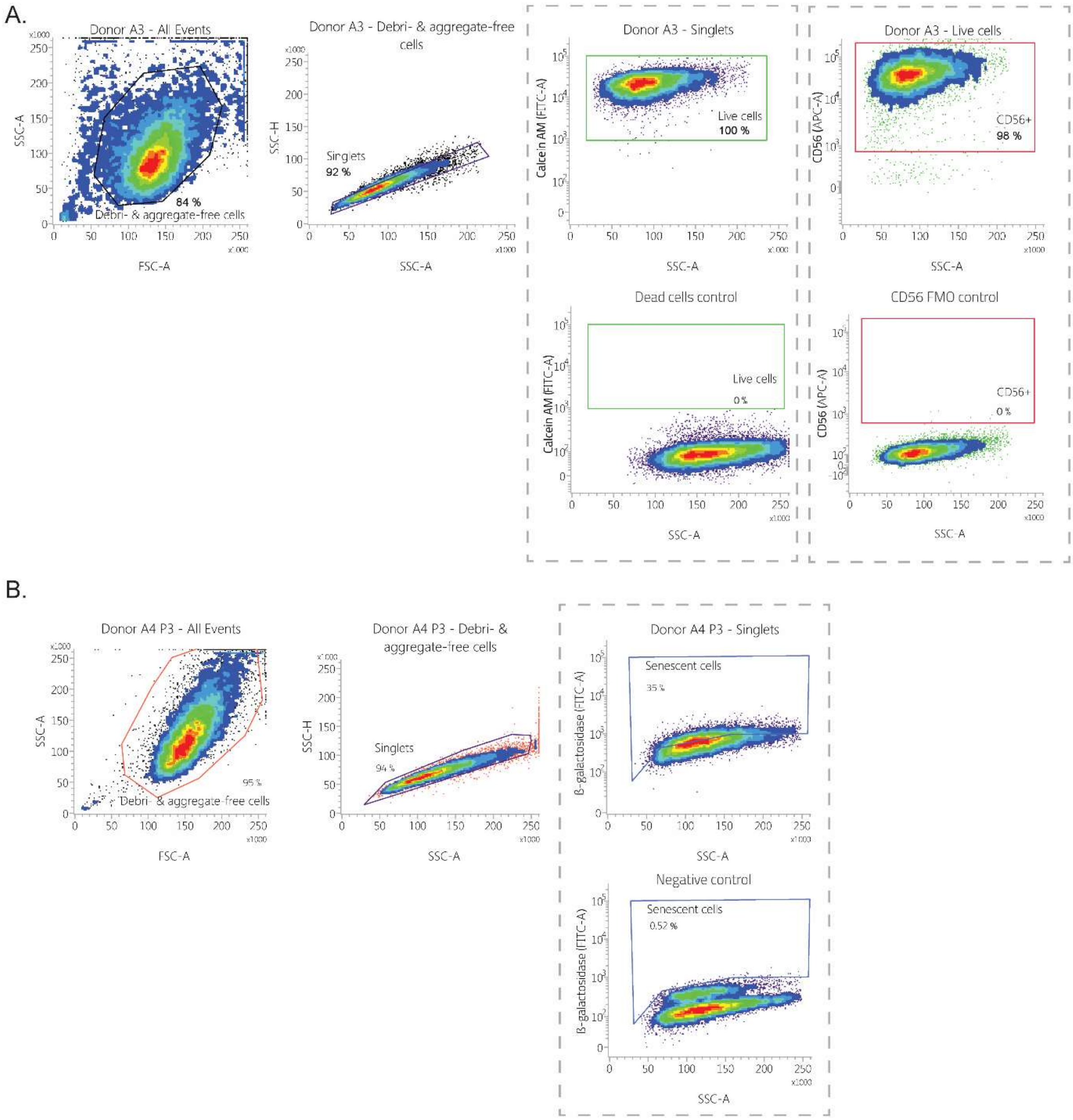

Supplementary Figure 2. PM+ medium supports sufficient expansion of bovine myoblasts, while retaining better cell morphology and differentiation capacity compared to PM, UM, and E8 media. **A.** Growth curves of myoblasts of three fetal donors expanded in the four different media on gelatin coated TCP. **B.** Maximum number of doublings reached by myoblasts (n=3) in different media before exceeding doubling time of 3 days. **C.** Morphology of myoblasts expanded in different media at different passages. White arrows indicate spontaneous differentiation into myotubes in PM and UM. Expansion in E8 medium led to formation of cell aggregates and subsequent detachment from the TCP before cells could reach P6. **D.** CD56 marker expression of myoblasts in different media (n=3). **E.** Fusion index of myoblasts expanded in different media (n=3). **F.** Representative immunofluorescence images of myotubes (tropomyosin in green) and cell nuclei (in blue) differentiated after expansion in different media at passages 1, 3, and 6. Box plots represent median ± IQR. FI = fusion index, P = passage, TCP = tissue culture plastic.

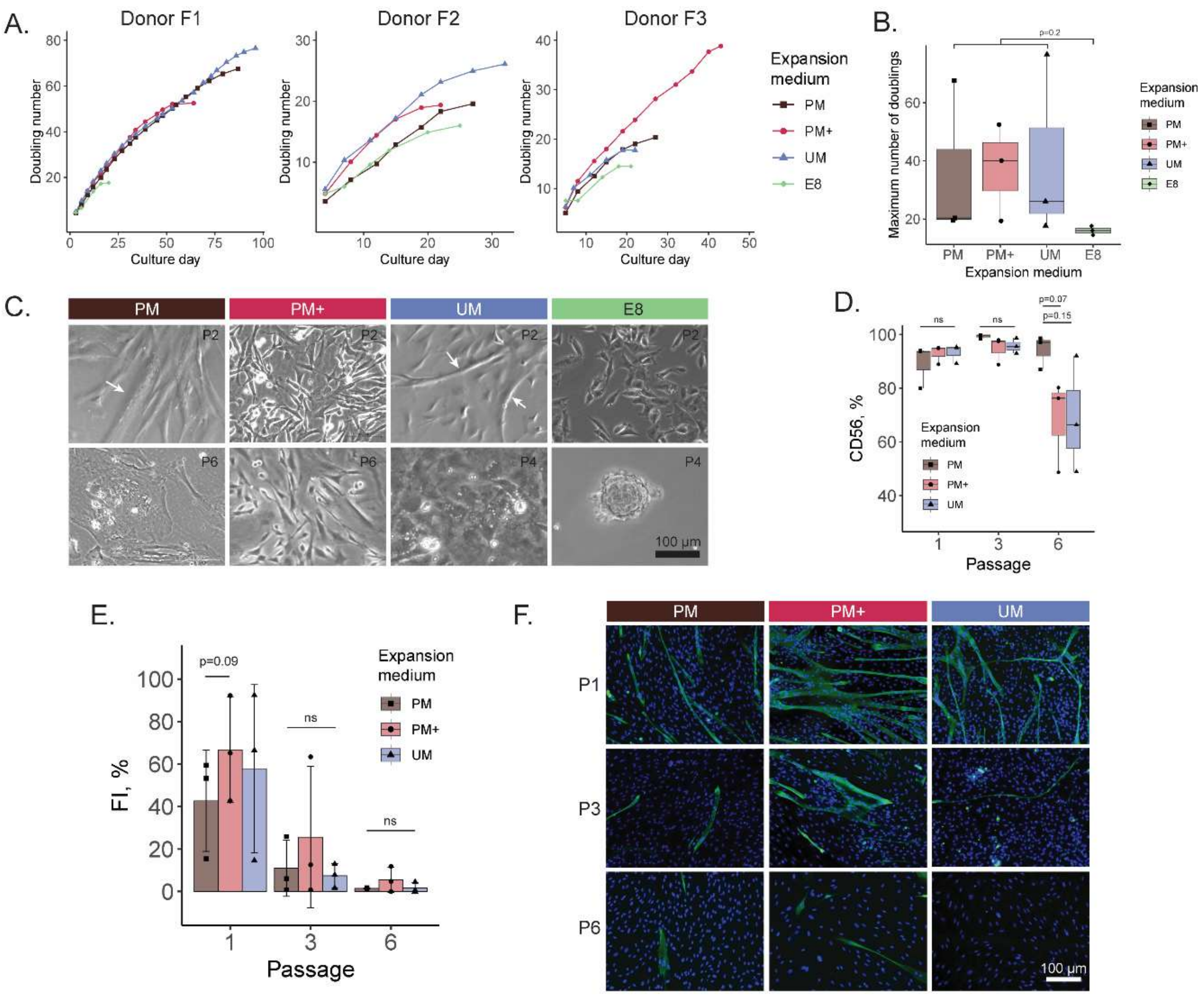

Supplementary Figure 3. Limited expansion and low FI of myoblasts cultured in E8 medium only, and E8 medium supplemented with 1 % BSA and/or on a Laminin521 (BioLamina, LN521) coated TCP. For Laminin521 coating, TCP was incubated with 10 µg/ml Laminin521 solution overnight at 4 °C. **A.** Growth curves of myoblasts of two donors expanded in different E8 medium conditions. **B.** Maximum number of doublings of myoblasts expanded in different E8 medium conditions (n=2). **C.** CD56 expression of myoblasts expanded in different E8 medium conditions at passage 3 (n=2). **D.** Fusion index of myoblasts expanded in different E8 medium conditions at passage 3 (n=2). **E.** Representative immunofluorescence images of myotubes (tropomyosin in green) and cell nuclei (in blue) differentiated after expansion in different E8 medium conditions at passage 3. Box plots represent median ± IQR. FI = fusion index, P = passage, TCP = tissue culture plastic.

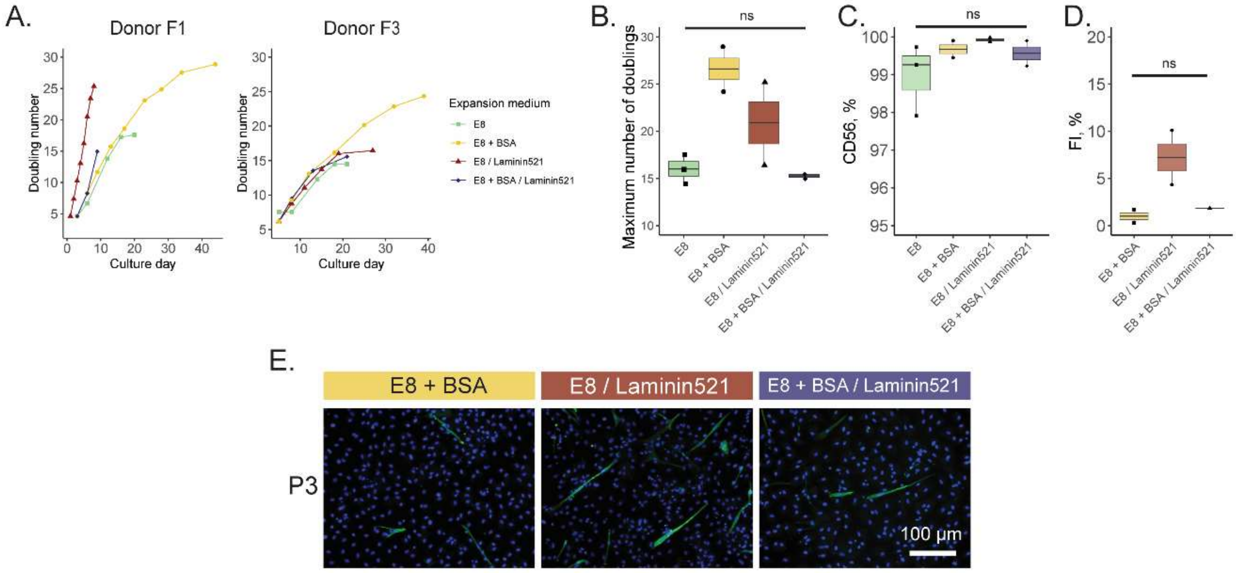

Supplementary Figure 4. Myogenic differentiation of bovine myoblasts in different differentiation medium formulations. Formation of myotubes was assessed with immunofluorescent staining for tropomyosin (in green) and cell nuclei (in blue), and quantified as Fusion Index (FI) using MyoFInDer software to determine the proportion of cell nuclei inside myotubes to the total number of nuclei. **A.** Representative images of fetal myoblasts differentiated in FM or SkFM media. **B.** FI of fetal myoblasts differentiated in FM or SkFM media (n=3). **C.** Representative images of fetal myoblasts differentiated in NL15, SFDM, or SkFM media. **D.** FI of fetal myoblasts differentiated in NL15, SFDM, or SkFM media (n=3). **E-H.** Effect of ERKi supplementation on adult myoblast differentiation (n=3-4) following the differentiation protocol reported by Eigler T. *et al.* (2021). ERKi was added either to PM or SkFM (1 µM final concentration) and compared to differentiation in SkFM. **I-L.** Effect of ERKi supplementation on adult myoblast differentiation (n=3-5) following the standard differentiation protocol used in our lab. ERKi was added either to PM or SkFM (1 µM final concentration) and compared to differentiation in SkFM. **M.** Comparison of the two differentiation protocols for assessment of ERKi supplementation during myoblast differentiation. Box plots represent median ± IQR. Lines connecting individual replicates represent donor pairing. FI = fusion index.

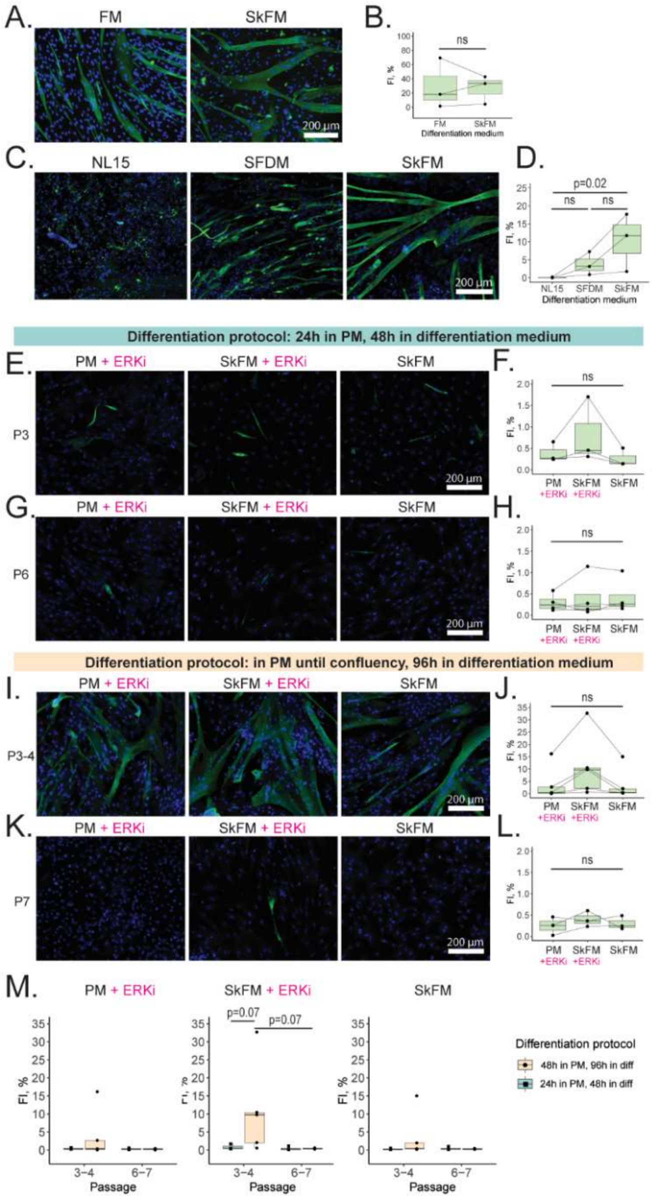

Supplement: Supplementary file 1 — Supplementary Information [file 42003_2025_9180_MOESM1_ESM.pdf]
