# Supplementary material for: Addressing hurdles in cultured meat by exploring reduced myogenesis after bovine myoblast expansion
Source: Commun Biol. 2025 Nov 27;8:1851. doi: 10.1038/s42003-025-09180-8 (PMC12748566; doi:10.1038/s42003-025-09180-8)
Supplement: Supplementary file 2 — Description of Additional Supplementary Materials [file 42003_2025_9180_MOESM2_ESM.pdf]

Description of Additional Supplementary Files

File name: Supplementary Data 1

Description: The source data behind the graphs in main figures of the paper

File name: Supplementary Data 2

Description: The source data behind the graphs in supplementary figures of the paper
