# Supplementary material for: Addressing hurdles in cultured meat by exploring reduced myogenesis after bovine myoblast expansion
Source: Commun Biol. 2025 Nov 27;8:1851. doi: 10.1038/s42003-025-09180-8 (PMC12748566; doi:10.1038/s42003-025-09180-8)
Supplement: Supplementary file 5 — Reporting summary [file 42003_2025_9180_MOESM5_ESM.pdf]

nature portfolio

Corresponding author(s): Lieven Thorrez

Last updated by author(s): Apr 29, 2025

Reporting Summary

Nature Portfolio wishes to improve the reproducibility of the work that we publish. This form provides structure for consistency and transparency in reporting. For further information on Nature Portfolio policies, see our Editorial Policies and the Editorial Policy Checklist.

Statistics

For all statistical analyses, confirm that the following items are present in the figure legend, table legend, main text, or Methods section.

n/a	Confirmed	
☐	☒	The exact sample size (*n*) for each experimental group/condition, given as a discrete number and unit of measurement
☐	☒	A statement on whether measurements were taken from distinct samples or whether the same sample was measured repeatedly
☐	☒	The statistical test(s) used AND whether they are one- or two-sided<br>*Only common tests should be described solely by name; describe more complex techniques in the Methods section.*
☐	☒	A description of all covariates tested
☐	☒	A description of any assumptions or corrections, such as tests of normality and adjustment for multiple comparisons
☐	☒	A full description of the statistical parameters including central tendency (e.g. means) or other basic estimates (e.g. regression coefficient) AND variation (e.g. standard deviation) or associated estimates of uncertainty (e.g. confidence intervals)
☐	☒	For null hypothesis testing, the test statistic (e.g. *F*, *t*, *r*) with confidence intervals, effect sizes, degrees of freedom and *P* value noted<br>*Give P values as exact values whenever suitable.*
☒	☐	For Bayesian analysis, information on the choice of priors and Markov chain Monte Carlo settings
☒	☐	For hierarchical and complex designs, identification of the appropriate level for tests and full reporting of outcomes
☐	☒	Estimates of effect sizes (e.g. Cohen's *d*, Pearson's *r*), indicating how they were calculated

Our web collection on statistics for biologists contains articles on many of the points above.

Software and code

Policy information about availability of computer code

Data collection	Microscopic fluorescence images were acquired with ZEN 3.0 (blue edition) (Carl Zeiss Microscopy GmbH, 2019) version 3.0.79.0000.<br>Microscopic color images were acquired using NDP.scan SQ 1.0.9 (Hamamatsu Photonics K.K.)<br>Cell counts were acquired using Countess automated cell counter (Invitrogen). Catalogue number C10281, serial number 10083-014.<br>Flow cytometry data was acquired with BD FACSuite v1.0.6.5230.<br>Genome sequencing was performed using NovaSeq S4. Demultiplexing and alignment was performed using Nucleotide Alignment Program.<br>Transctiptome analysis was performed using HiSeq 4000 (Illumina). Quality control of raw reads was performed with FastQC v0.11.7, and the adapters were filtered using Trimmomatic v0.39.<br>Proteomic analysis was carried out using LC-MS/MS Ultimate 3000 RSLCnano system in-line connected to a Q Exactive HF mass spectrometer (Thermo).
Data analysis	MyoFInDer software was used for processing of images of myoblast fusion (all documentation and links to download can be found in an open-access publication: 10.1089/ten.TEA.2024.0049).<br>QIAGEN's Ingenuity Pathway Analysis (IPA) software (version 111725566) was used to find significantly differentially expressed pathways.<br>The rest of data analysis was performed using RStudio version 4.3.1.

For manuscripts utilizing custom algorithms or software that are central to the research but not yet described in published literature, software must be made available to editors and reviewers. We strongly encourage code deposition in a community repository (e.g. GitHub). See the Nature Portfolio guidelines for submitting code & software for further information.

Data

Policy information about availability of data

All manuscripts must include a data availability statement. This statement should provide the following information, where applicable:

- Accession codes, unique identifiers, or web links for publicly available datasets
- A description of any restrictions on data availability
- For clinical datasets or third party data, please ensure that the statement adheres to our policy

Data analysis scripts for the proteome and transcriptome analysis were made available via Zenodo repository (DOI: 10.5281/zenodo.13897183). Raw and processed mass spectrometry data for proteome analysis can be accessed under PXD052959. Any additional raw or processed data are available upon request.

Research involving human participants, their data, or biological material

Policy information about studies with human participants or human data. See also policy information about sex, gender (identity/presentation), and sexual orientation and race, ethnicity and racism.

Reporting on sex and gender	Not applicable
Reporting on race, ethnicity, or other socially relevant groupings	Not applicable
Population characteristics	Not applicable
Recruitment	Not applicable
Ethics oversight	Not applicable

Note that full information on the approval of the study protocol must also be provided in the manuscript.

Field-specific reporting

Please select the one below that is the best fit for your research. If you are not sure, read the appropriate sections before making your selection.

☒ Life sciences ☐ Behavioural & social sciences ☐ Ecological, evolutionary & environmental sciences

For a reference copy of the document with all sections, see nature.com/documents/nr-reporting-summary-flat.pdf

Life sciences study design

All studies must disclose on these points even when the disclosure is negative.

Sample size	Experiments with in vitro cultured primary cells are laborious and resource intensive. Usually, the minimum sample size of 3 donors is reported. Our study presents results from 5 adult and 5 fetal bovine donors, as a compromise between higher statistical power and limited resources.<br>Additional experiments were presented in the supplementary material with various sample sizes (n=2-5). No power tests were performed to determine the right sample size, but rather a few donors were tested to help selection of the culture conditions for further experiments presented in the study.
Data exclusions	No data were excluded from this study.
Replication	Some of the variables (such as cell doubling numbers, fusion index, CD56 marker analysis) were analyzed independently for the same samples in other unrelated experiments are were showing similar results. Additionally, the instruments used for data acquisition are regularly maintained and pass quality controls.
Randomization	Bovine donors for muscle tissue collection were randomly selected and were only based on availability at the local abattoir.
Blinding	The investigators were not blinded during data collection and analysis to avoid mistakes during the execution of the experiments. Potential bias was minimized by using data processing softwares (e.g. image analysis, flow cytometry).

Reporting for specific materials, systems and methods

We require information from authors about some types of materials, experimental systems and methods used in many studies. Here, indicate whether each material, system or method listed is relevant to your study. If you are not sure if a list item applies to your research, read the appropriate section before selecting a response.

Materials & experimental systems

n/a	Involved in the study
☐	☒ Antibodies
☐	☒ Eukaryotic cell lines
☒	☐ Palaeontology and archaeology
☒	☐ Animals and other organisms
☒	☐ Clinical data
☒	☐ Dual use research of concern
☒	☐ Plants

Methods

n/a	Involved in the study
☒	☐ ChIP-seq
☐	☒ Flow cytometry
☒	☐ MRI-based neuroimaging

Antibodies

Antibodies used	1. Mouse monoclonal IgG2a APC-conjugated anti-CD56 antibody (MEM-188 clone, Invitrogen, MA1-19462)<br>2. Mouse IgG1 monoclonal anti-tropomyosin (sarcomeric) antibody (clone CH1, Sigma-Aldrich, T9283)<br>3. Goat anti-mouse polyclonal IgG (H+L) highly cross-adsorbed secondary antibody conjugated with Alexa Fluor 488 (Invitrogen, A-11029)
Validation	1. MEM-188 clone of CD56 antibody was validated for use in bovine myoblasts according to existing publication (DOI: 10.1038/s41598-018-28746-7)<br>2. Tropomyosin antibody was validated for staining bovine myotubes in this study. Negative controls, where the myotubes were only incubated with a secondary fluorescent antibody (see antibody #3), failed to show any fluorescence. When anti-tropomyosin primary antibody and the secondary antibody were used, myotubes (also visible through phase contrast filters), but not the surrounding cells, were brightly fluorescent.<br>3. No unspecific binding of goat anti-mouse Alexa Fluor 488 secondary antibody to bovine cells was detected, as evidenced by the absence of green fluorescence.

Eukaryotic cell lines

Policy information about cell lines and Sex and Gender in Research

Cell line source(s)	All primary bovine myoblasts were isolated from biopsies of biceps femoris muscle of Belgian Blue cattle obtained at the local abattoir.<br><br>Fetal donors:<br>Donor F1: male<br>Donor F2: male<br>Donor F3: male<br>Donor F4: female<br>Donor F5: male<br><br>Adult donors:<br>Donor A1: female<br>Donor A2: female<br>Donor A3: female<br>Donor A4: male<br>Donor A5: female
Authentication	None of the cell lines were authenticated.
Mycoplasma contamination	Cell lines used in this study were tested for mycoplasma contamination with MycoAlert Detection Kit (Lonza) and were confirmed to be negative.
Commonly misidentified lines<br>(See ICLAC register)	Not applicable

Plants

Seed stocks	Not applicable
Novel plant genotypes	Not applicable
Authentication	Not applicable

Flow Cytometry

Plots

Confirm that:

☒ The axis labels state the marker and fluorochrome used (e.g. CD4-FITC).

☒ The axis scales are clearly visible. Include numbers along axes only for bottom left plot of group (a 'group' is an analysis of identical markers).

☒ All plots are contour plots with outliers or pseudocolor plots.

☒ A numerical value for number of cells or percentage (with statistics) is provided.

Methodology

Sample preparation	1. The proportion of CD56+ cells, representing myoblasts, in isolated cells was characterized using CD56 immunolabeling and flow cytometric analysis. After passaging, 7.5 x10^5 cells were taken for CD56 marker analysis. After washing the cell suspension in 10 % FBS in PBS, 2.5 x10^5 of the cells were incubated in PE anti-human CD56 antibody (Invitrogen, MA1-19462) diluted 1:20 in 10 % FBS in PBS, for 25 min on ice in the dark. Another 2.5 x10^5 cells were incubated in the absence of the antibody and used as the Fluorescence Minus One (FMO) control. Lastly, 2.5 x10^5 cells were killed by incubation for 10 min at 65 °C to be used as a control for gating out dead cells. After incubation steps, the cell suspensions were washed 2x in 3 % FBS in PBS and resuspended in 100 nM calcein (Anaspec, AS-89201) solution. After 20 min, the CD56+ cells were determined using a FACSVerse™ (BD Biosciences) flow cytometer. At least 10 000 debri- and aggregate-free cells were measured.<br><br>2. Quantitative analysis of senescence was carried out with the CellEvent™ Senescence Green Flow Cytometry Assay Kit (Invitrogen, C10850) following the manufacturer's protocol. In short, 5 x10^5 cells were washed and then fixed in 2 % formaldehyde for 10 min at room temperature. After 2 washing steps in 1 % BSA, the cells were resuspended in CellEvent™ Senescence Green Probe diluted 1:3000 in CellEvent™ Senescence Buffer and incubated at 37 °C for 1 h in the dark. The negative control consisted of cells incubated in the buffer without the Green Probe. After incubation, the samples were washed in 1 % BSA and measured using a FACSVerse™ flow cytometer (BD Biosciences). Ten thousand debris- and aggregate-free events were further gated to isolate singlets. The singlets were then gated in the green fluorescent channel using the negative control to identify the percent of senescent cells.
Instrument	BD FACSVerse Flow Cytometer, catalogue number 651155, serial number Z6511550336<br><br>CytoFlex S flow cytometer (Analis NV)
Software	BD FACSuite v1.0.6.5230<br><br>CytExpert version 2.4.0.28
Cell population abundance	Details provided in Supplementary Figure 1.
Gating strategy	Details provided in Supplementary Figure 1.

☒ Tick this box to confirm that a figure exemplifying the gating strategy is provided in the Supplementary Information.
